# Cortical Pathology in RRMS: Taking a Cue from Four Sisters

**DOI:** 10.1155/2012/760254

**Published:** 2012-09-27

**Authors:** Massimiliano Calabrese, Dario Seppi, Eleonora Cocco, Valentina Poretto, Francesca Rinaldi, Paola Perini, Paolo Gallo

**Affiliations:** ^1^The Multiple Sclerosis Centre of Veneto Region, First Neurology Clinic, Department of Neurosciences, University Hospital of Padua, Via Giustiniani 5, 35128 Padoua, Italy; ^2^Department Public Health Clinical and Molecular Medicine, University of Cagliari, 09124 Cagliari, Italy

## Abstract

*Background*. Although grey matter pathology is a relevant aspect of multiple sclerosis (MS) both with physical and cognitive rebounds, its pathogenesis is still under investigation. To what extent the familial and sporadic cases of MS differ in cortical pathology has not been elucidated yet. Here we present a multiple case report of four sisters affected by MS, all of them having a very high burden of cortical pathology. *Methods*. The clinical and grey matter MRI parameters of the patients were compared with those of twenty-five-aged matched healthy women and 25 women affected by sporadic MS (matched for age, disease duration, EDSS, and white matter lesion load). *Results*. Despite their short disease duration (<5 years), the four sisters showed a significant cortical thinning compared to healthy controls (*P* = 0.003) and sporadic MS (*P* = 0.041) and higher CLs number (*P* < 0.001) and volume (*P* < 0.001) compared to sporadic MS. *Discussion*. Although limited to a single family, our observation is worth of interest since it suggests that familial factors may account for a peculiar involvement of the cortex in MS pathology. This hypothesis should be further evaluated in a large number of multiplex MS families.

## 1. Introduction

Multiple sclerosis (MS) is an autoimmune chronic inflammatory disease of the central nervous system whose etiopathogenesis is believed to be the result of a complex interaction of genetic and environmental factors [1, 2]. Beside the extensive inflammatory demyelination observed in the white matter (WM), the involvement of grey matter (GM), that is, focal lesions [3] and atrophy [4, 5], is a well-known feature of MS pathology [6]. Several magnetic resonance imaging (MRI) studies have demonstrated that a significant cortical damage is a frequent finding in all MS phenotypes [3] (including more than one-third of individuals at disease onset and also in patients with radiological isolated syndrome [7]), sometimes preceding the appearance of any WM lesion [8, 9]. Moreover, GM damage was found to have a significant impact on clinical [10–12] and cognitive [13, 14] disability as longitudinal studies have disclosed a more severe prognosis in patients having a high degree of cortical damage [10, 15]. Thus, cortical pathology has to be considered a further element of heterogeneity, and its use for a better clinical stratification of MS patients is currently under evaluation. 

Up to date, no study has been performed to evaluate the degree of cortical pathology in patients from multiplex MS families and to assess whether cortical pathology might be a “familial share factor” that could be used for prognostic purposes in familial cases versus sporadic cases. We describe four sisters affected by relapsing remitting MS (RRMS), all characterized by a particularly severe focal and diffuse cortical damage despite their short disease duration. 

## 2. Methods

### 2.1. Patients

While conducting a study on cortical pathology in MS, we came upon 4 sisters, aged 43, 46, 48, and 49 years, all affected from relapsing remitting MS (RRMS), according to the Polman's diagnostic criteria [16] and currently followed in our MS Centre. The sisters and all their parents and grandparents were Caucasian and original from the Veneto Region of Italy. No other case of MS or others autoimmune diseases were reported among the first-degree relatives. Each patient underwent neurological examination (Expanded Disability Status Scale, EDSS) and high-resolution brain and spinal cord MRI. Clinical and instrumental data are summarized in Table 1. MRI examination was also obtained from 25 women affected by sporadic RRMS (matched for age, disease duration, EDSS, and T2-WM lesion load) and 25 healthy women (matched for age).

#### 2.1.1. Clinical History

**Sister 1 (aged 49)**

Her history began in 2007 with a facial palsy that was not recognized, at that time, as the MS onset. Only in 2011, when the patient developed an obvious incomplete paralysis of any eye movement, she underwent MRI examination and cerebrospinal fluid (CSF) analysis that both confirmed the diagnosis of MS. In the same year she started a disease-modifying drug (Interferon beta 1b).

**Sister 2 (aged 48)**

She complained in 2006 impaired discrimination of sharp/dull at the right arm. A diagnosis of “possible Multiple Sclerosis” was suggested at that time on the base of dissemination in space of the lesions. The patients had more than 9 periventricular white matter lesions and the presence of IgG oligoclonal bands in CSF. During following years, the patients underwent a clinical and radiological follow-up until 2008 when, in absence of any relapse, the appearance of a new demyelinating lesion, enhanced by gadolinium allowed the confirmation of “definite Multiple Sclerosis.” A therapy based on Interferon beta 1a was started few months after the diagnosis of MS. Between 2008 and 2010, she showed 3 new clinical relapses and in the early 2011 she has been shifted from Interferon beta 1a to Natalizumab. In the last year no new relapses or new MRI lesions have been observed.

**Sister 3 (aged 46)**

In 2009, she complained a large scotoma in right eye associated to obvious disc pallor and suggestive of optic neuritis. MRI examinations showed multiple demyelinated lesions, some of them hypointense in T1-weighted sequences and 2 of these enhanced by gadolinium. IgG oligoclonal bands in CSF were demonstrated and the visual evoked potentials showed alterations consistent with demyelination in both eyes. Her condition has been defined as a “Multiple Sclerosis” according to the recent revision diagnostic criteria [17].

**Sister 4 (aged 43)**

In her childhood she suffered from febrile convulsions; in 2009 following several migraine episodes, she underwent an MRI examination, which showed multiple T2 hyperintense brain lesions suggestive of a demyelinating disease. At that time her neurological examination was normal and therefore a diagnosis of “radiological isolated syndrome” was indicated. In 2010, she suffered from acute hyposthenia of the right leg associated to urge incontinence. A new MRI showed the appearance of 4 contrast-enhancing lesions. Notwithstanding the beginning of interferon beta 1a in the early 2011 she complained 2 severe relapses during 2011 and was shifted to Natalizumab in September 2011.

All demographic and clinical characteristics of these patients at study entry are summarized in Table 1.

### 2.2. Image Acquisition Protocol

All images were acquired using a 1.5 T scanner (Achieva, Philips Medical Systems, Best, The Netherlands) with 33 mT/m power gradient and a 16-channel head coil. No major hardware upgrades of the scanner occurred during the study period and bimonthly quality assurance sessions took place to guarantee measurement stability. The following images were acquired from each subject: (1) *double inversion recovery (DIR)*—repetition time (TR) = 15631 msec, echo time (TE) = 25 msec, inversion time (TI) = 3400 msec, delay = 325 msec, echo train length (ETL) = 17, 50 contiguous axial slices with a thickness = 3 mm, a matrix size = 130 × 256, and a field of view (FOV) = 250 × 200 mm^2^; (2) *fluid-attenuated inversion recovery (FLAIR)*—TR = 10000 msec, TE = 120 msec, TI = 2500 msec, ETL = 23, 50 contiguous axial slices with a thickness = 3.0 mm, a matrix size = 172 × 288, and a FOV = 250 × 200 mm^2^; (3) three V*olumetric fast-field echo (FFE) sequences*—120 contiguous axial slices, TR = 25 msec, TE = 4.6 msec, flip angle = 30°, slice thickness = 1.0 mm, matrix size = 256 × 256, and a FOV = 250 × 250 mm^2^ were acquired. The last one was acquired 5 minutes after gadolinium-EDTA (0.1 mmol/kg) intravenous administration; (4) *cervical and dorsal cord short time inversion recovery*—TR = 2500 msec, TE = 60 msec, inversion time = 170 msec; 15 sagittal slices, slice thickness = 3.5 mm, gap = 0 mm, matrix size = 272 × 512, and filed of view = 250 × 250 mm^2^.

### 2.3. Image Analysis

**(a) Cortical Lesion Number and Volume**

All images were analysed at the Advanced Neuroimaging Laboratory of the Multiple Sclerosis Centre in Padoua, by consensus of two experienced observers and in a blind fashion. The number of CLs was assessed on DIR images (Figures 2 and 3) following the recent recommendations for CLs scoring in patients with MS [18].

The CL volume was calculated using a semiautomatic thresholding technique based on Fuzzy C-mean algorithm [19] included in Medical Images Processing, Analysis and Visualization tool (MIPAV) (http://mipav.cit.nih.gov/).

**(b) WM Lesion Number and Volume**

The same procedure was applied to FLAIR images, to identify and segment WM lesions, thus obtaining a T2 hyperintense WM lesion volume (T2WMLV) at baseline and followup. The number of contrast enhancing lesions and spinal cord lesions was also evaluated.

**(c) Cortical Thickness Evaluation**

Global cortical thickness (CTh) (mean of right and left hemispheres) was performed, as previously described [20] on the volumetric FFE data sets by means of Freesurfer image analysis [20] suite (release v5.1.0), available online (http://surfer.nmr.mgh.harvard.edu/). All images were accurately controlled for errors/artifacts by an experienced neurologist (MC). A first visual  inspection was necessary after the skull stripping to visualize areas not completely removed, while a second visual inspection was needed after WM/GM segmentation, to visualize possible misclassifications (especially due to MS lesions).

### 2.4. Statistical Analysis

A Fisher exact and Mann-Whitney tests were used to compare familial cases with RRMS and healthy subjects with respect to demographic and clinical characteristics. All statistical analyses were performed using SPSS v.18 and R, a statistical package available at http://www.r-project.org/.

## 3. Results

**Figure 1:**
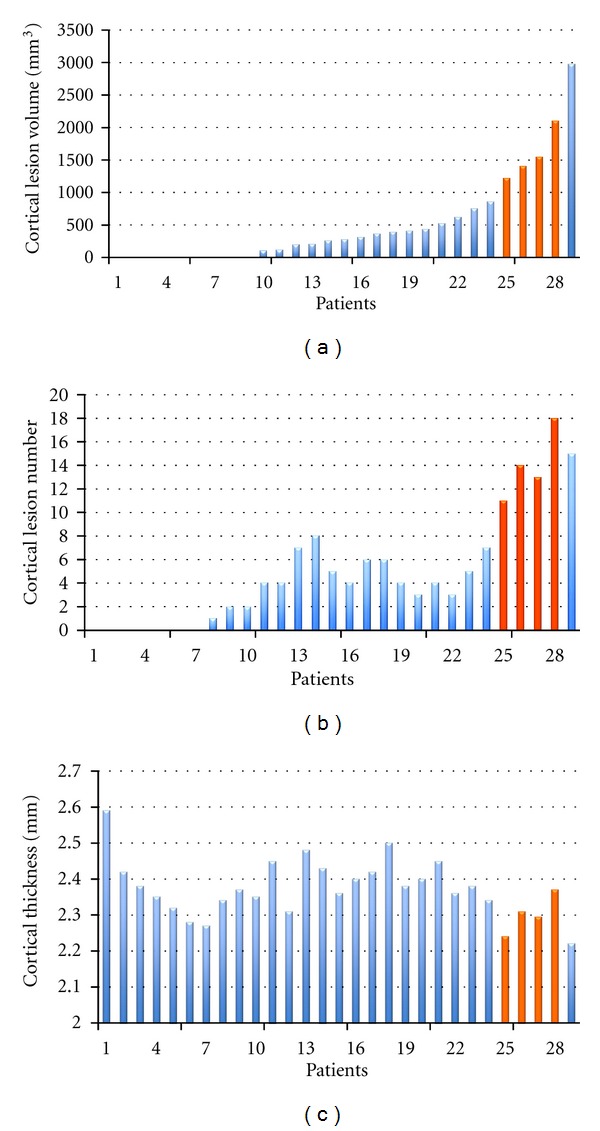
Histograms of distribution of cortical lesion volume and number and cortical thickness of the entire MS population. Patients are ordered on the base of cortical lesion volume. Orange bars highlighted the values of the 4 sisters.

The distribution of cortical lesion number and volume and cortical thickness in the entire population are described in Figure 1.

**Figure 2:**
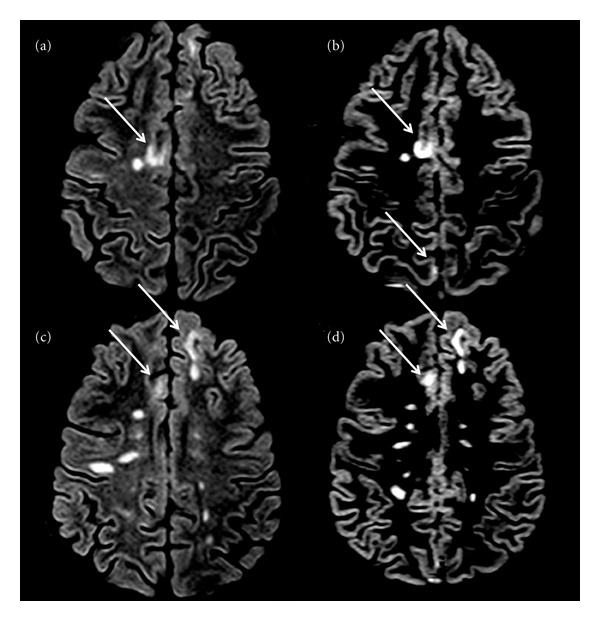
Axial DIR of ((a), (b)) sister 1 and ((c), (d)) sister 2. Several cortical lesions (arrows) and white matter lesions are depicted.

**Figure 3:**
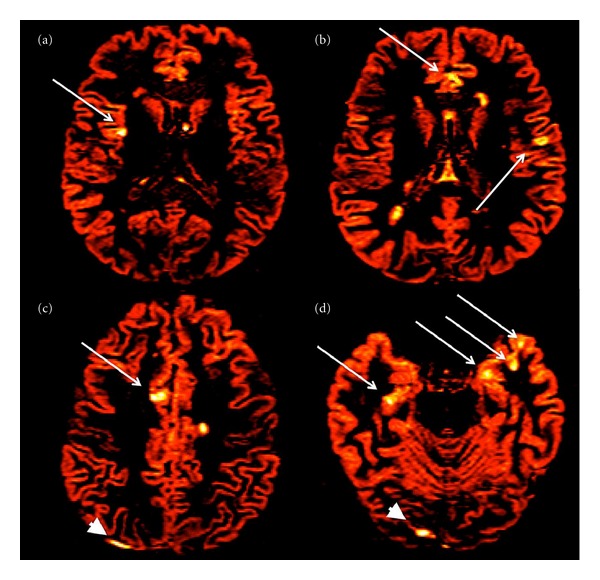
Red-coloured axial DIR of ((a), (b)) sister 3 and ((c), (d)) sister 4. Several cortical lesions (arrows), white matter lesions, and some artefacts (arrowheads) are depicted.

A significant global cortical thinning was observed in the 4 sisters compared to both the healthy women (*P* = 0.003) and the sporadic RRMS females (*P* = 0.041). The regional analysis of cortical thinning could not be performed given the low statistical power of our sample size. The CLs number (*P* < 0.001) and the total CL volume (*P* < 0.001) were higher in the sisters (Figures 2 and 3) than in the sporadic RRMS cases, while no significant difference was observed in the number of WM lesions, in the T2WM lesion volume, in the number of gadolinium-enhancing lesions and in spinal cord lesions (Table 2). 

## 4. Discussion

We describe four sisters affected from RRMS whose peculiarity was that all had an unexpectedly severe focal and diffuse cortical pathology despite their quite short disease duration (<5 years). Both number and volume of cortical lesions appeared extraordinary high in these sisters when compared to the sporadic RRMS patients, especially considering the almost identical T2WM lesion load, the number of contrast enhancing lesions and spinal cord lesions.

The analysis of cortical thickness confirmed that these four patients were also characterized by a widespread cortical damage. Indeed, their global cortical atrophy reached the significance when compared not only to healthy females, but, more interestingly, when compared to sporadic RRMS cases having the same age and disease duration. This finding is even more relevant if we consider the extremely low statistical power of our sample size.

Our observation is interesting since it describes a cluster of severe cortical pathology in a multiplex MS family. We are aware that this is only a descriptive case and that only preliminary consideration can rise from these data. However, since occasional observations may help in designing working hypothesis, we would hypothesize that the presence of such a severe cortical damage in four members of the same family cannot be casual, but possibly the expression of a familial-related predisposition to develop a grey-matter rather than a white-matter oriented inflammation. So far, only a few studies have tried to correlate familial-shared factors to the severity of the structural changes observed by MRI in the white matter of MS patients. Namely, a relationship was found between some HLA-alleles and the severity of white matter pathology detected by means of conventional and unconventional neuroimaging [21–23]. Moreover, genes could potentially be involved in cortical demyelination, as observed in the autoimmune encephalomyelitis [24], but no data exists in human. 

To what extent environmental factors may also play a role in favouring the development of MS in several members of the same family remains an open question. Indeed, this possibility cannot be excluded since cortical lesions were found to be strictly associated with meningeal inflammation [6, 25] and a role for the EBV has been recently suggested [26].

Recent pathological [25] and MRI [3–5] studies have disclosed that cortical pathology is relevant in MS and may be demonstrated in very early disease phases [3–9, 20]. However, although cortical lesions may appear before white matter lesions [3, 8, 9, 25], their load is usually low at the beginning of the disease and progressively increases [10], reaching the highest values in the secondary progressive phase [3]. For this reason, the four sisters here described, given their short disease duration, constitute a particularly stimulating cue to further analyse the extension and severity of cortical pathology in a larger number of multiplex MS families. 

## Figures and Tables

**Table 1:** Demographic, clinical, and MRI characteristics of four sisters affected with multiple sclerosis.

	Sister 1	Sister 2	Sister 3	Sister 4
Age (years)	49	48	46	43
Disease duration (years)	5	6	3	2
Type of onset	Brainstem	Brainstem	Optical nerve	Spinal cord
VEP	+	+	+	n.d
BOIgG	+	+	+	+
EDSS	3.0	2.0	1.0	3.0
Treatment	IFN beta 1b (since 2008)	Natalizumab (since 2012)	IFN beta 1a (since 2010)	Natalizumab (since 2012)
Relapses in the last 2 years	2	2	1	3
WM brain lesions	6	15	10	16
SCL	0	0	0	1
CEL	0	0	0	0
CLs	11	14	18	13
CL volume (mm^3^)	1225	1540	2100	1420
Global CTh (mm)	2.31 ± 0.5 (1.8–3.2)	2.37 ± 0.6 (1.8–3.1)	2.24 ± 0.5 (1.6–3.0)	2.30 ± 0.7 (1.9–3.3)

VEP: visual evocated potentials; BOIgG: oligoclonal bands; EDSS: expanded disability status scale; WM: white matter; SCL: spinal cord lesions; CEL: contrast enhancing lesion; CL: cortical lesion; CTh: cortical thickness.

**Table 2:** Clinical and MRI characteristics of the studied populations.

	Familial cases (*n* = 4)	Sporadic RRMS (*n* = 25)	Healthy subjects (*n* = 25)
Age (years)	46.5 ± 2.6 (43–49)	46.5 ± 2.8 (42–49)	46.5 ± 2.8 (41–50)
Disease duration (years)	4.0 ± 1.8 (2–6)	4.1 ± 2.2 (1–8)	n.a.
EDSS	2.5 (1.0–3.0)	2.5 (1.0–4.0)	n.a.
WM lesions number	11.7 ± 4.6 (6–16)	11.4 ± 7.5 (4–28)	0
T2WMLV (cm^3^)	6.1 ± 4.2 (2.1–11.4)	6.3 ± 5.8 (3.2–12.1)	0
Patients with CEL	0	4 (16%)	0
Patients with SCL	1 (25%)	9 (36%)	0
CLs number	14.0 ± 2.9 (11–18)^$^	3.6 ± 3.6 (0–15)	0
CL volume (mm^3^)	1571 ± 376 (1225–2100)^$^	352 ± 602 (0–2975)	0
Global CTh (mm)	2.30 ± 0.05 (2.24–2.37)^∗$^	2.38 ± 0.07 (2.22–2.59)	2.47 ± 0.07 (2.12–2.69)

*P < 0.05 versus healthy subjects; ^$^ *P* < 0.05 versus sporadic RRMS.

EDSS: expanded disability status scale; T2WMLV: T2 white matter lesion volume; WM: white matter; CEL: contrast enhancing lesion; SCL: spinal cord lesions; CL: cortical lesion; CTh: cortical thickness.
